# Class-C Linearized Amplifier for Portable Ultrasound Instruments

**DOI:** 10.3390/s19040898

**Published:** 2019-02-21

**Authors:** Hojong Choi

**Affiliations:** Department of Medical IT Convergence Engineering and Kumoh National Institute of Technology, Gumi 39253, Korea; hojongch@kumoh.ac.kr

**Keywords:** class-C amplifier, transistor linearizer, portable ultrasound instruments

## Abstract

Transistor linearizer networks are proposed to increase the transmitted output voltage amplitudes of class-C amplifiers, thus, increasing the sensitivity of the echo signals of piezoelectric transducers, which are the main components in portable ultrasound instruments. For such instruments, class-C amplifiers could be among the most efficient amplifier schemes because, compared with a linear amplifier such as a class-A amplifier, they could critically reduce direct current (DC) power consumption, thus, increasing the battery life of the instruments. However, the reduced output voltage amplitudes of class-C amplifiers could deteriorate the sensitivity of the echo signals, thereby affecting the instrument performance. Therefore, a class-C linearized amplifier was developed. To verify the capability of the class-C linearized amplifier, typical pulse-echo responses using the focused piezoelectric transducers were tested. The echo signal amplitude generated by the piezoelectric transducers when using the class-C linearized amplifier was improved (1.29 V_p-p_) compared with that when using the class-C amplifier alone (0.56 V_p-p_). Therefore, the class-C linearized amplifier could be a potential candidate to increase the sensitivity of echo signals while reducing the DC power consumption for portable ultrasound instruments.

## 1. Introduction

Ultrasound instruments have been used widely to obtain anatomical information from targets in automotive, semiconductor, structural health monitoring, renewable energy, and medical applications [1,2,3]. In particular, portable ultrasound instruments are recently highlighted as medical instruments used in ambulances and emergency rooms because they provide real-time, nonionizing, and noninvasive characteristics for patients’ diagnosis before utilizing other invasive medical instruments, such as X-ray, computed tomography, and positron-emission tomography to obtain the structural and physiological information [2]. 

The ultrasound instrument performance when using array transducers is originally affected by the nonlinear acoustic properties, thus generating grating lobes and speckle patterns in the imaging [4,5]. Additionally, portable ultrasound instruments suffer from unwanted heat generated by the large battery consumption of the transmitter [2]. Therefore, efficient battery management is one of key factors to evaluate portable ultrasound instruments. To reduce the battery consumption, the direct current (DC) power consumption needs to be reduced while sustaining reasonable performance, as the DC power consumption could be crucial problem to be handled by amplifier performance. Similar to conventional ultrasound instruments, portable ultrasound instruments are composed of a transmitter, piezoelectric transducer, and receiver [6]. 

The most DC power consumption comes from the amplifier in the transmitter’s analog-to-digital converter and the digital-to-analog converter in the receiver, respectively [6]. The piezoelectric transducer is the most important electromechanical device to produce the acoustic or electrical waveforms in the instruments [7]. The amplifier triggers the piezoelectric transducers, generating acoustic waveforms, and then the reflected acoustic waveforms are converted into electrical signals by the piezoelectric transducers [8,9]. Therefore, the amplifier is also a crucial design factor for the portable ultrasound instruments. Compared with linear amplifiers, nonlinear amplifiers have been shown to reduce DC power consumption in ultrasound instruments [4,10,11]. However, the reduced signal amplitudes of the echo signals caused by the nonlinear amplifiers have limited widespread use of portable ultrasound machines because of low sensitivity. To reduce signal loss, a proper nonlinear amplifier design is very important because the amplifier is a last-stage electronic component in the transmitter to excite the piezoelectric transducers [6]. 

Several nonlinear amplifiers have been developed for ultrasound applications. A push–pull class-B amplifier was implemented for the 50-kHz ultrasonic transducer [12]. Class-D amplifiers were developed for 41.27-kHz Langevin sample transducer and high-power piezoelectric load [13,14]. The class-E amplifier was used for a 40.25 kHz inductive piezoelectric transducer [15]. The improved performance of these nonlinear amplifiers could enhance the piezoelectric transducer performance if the transmit output signals generated by the nonlinear amplifiers are improved. Additionally, the class-C amplifier is one of the most efficient amplifiers among the nonlinear amplifiers; thus, it could be useful to minimize unwanted heat generation [16,17]. However, the class-C amplifier suffers from nonlinear operations due to low DC operating points. Therefore, a linearizer scheme to increase the voltage gain for class-C amplifiers could be useful to improve the piezoelectric transducers for portable ultrasound instruments. 

**Figure 1:**
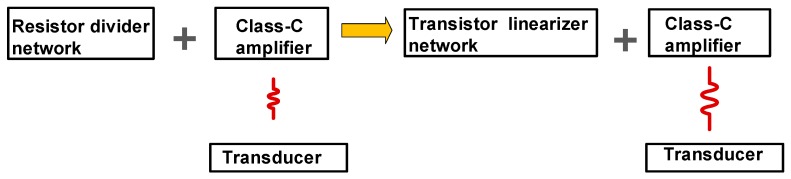
Concept of the class-C linearized amplifier for portable ultrasound instruments.

Figure 1 shows the concept of the class-C linearized amplifier for portable ultrasound instruments. The amplifier typically uses the resistor divider network to bias the DC voltages for amplifier operations [18]. However, high-voltage environment affects the bias voltages of the resistor divider network such that it can affect the output performance variance of the amplifier [19]. Additionally, the piezoelectric transducer component itself is capacitive-type device such that the nonlinear behavior under a high-voltage environment is related to the amplifier performance [20]. In particular, class-C amplifiers are critically affected by the DC bias voltages due to low DC operating points. Therefore, a transistor linearizer, dedicated to improve the class-C amplifier output performance, was developed by stabilizing the DC bias voltages under high-voltage environments. 

For the amplifier design, the expected simulation libraries of the power metal–oxide–semiconductor field-effect transistors (MOSFETs) do not have signal-distortion accuracy for the sub-decibel level [21]. Additionally, the temperature model parameters for power MOSFETs in the simulation tool are sometimes unpredictable under high-voltage environments [22]. For power MOSFETs, hot-carrier injection effects generate inaccurate gate-source voltage variances under high-voltage environments [23]. Therefore, the amplifier design needs to be started at the hands-on printed circuit board level to produce proper amplifier performance. Section 2 describes the schematic diagrams and operating mechanisms of the class-C amplifier with a transistor linearizer scheme. Section 3 shows the measured results of the class-C amplifier with the resistor divider network and transistor linearizer network, including pulse-echo responses using the piezoelectric transducer. Section 4 provides the concluding remarks of the paper. 

## 2. Materials and Methods

**Figure 2:**
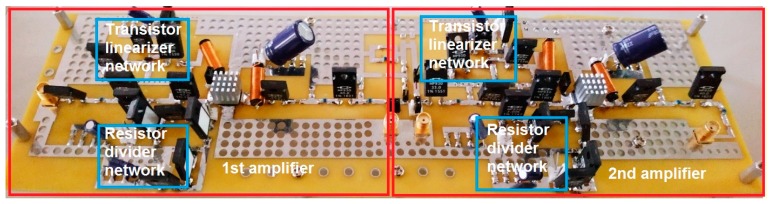
Photo of the class-C amplifiers with resistor divider network and transistor linearizer network.

Figure 2 shows the fabricated printed circuit board of the class-C amplifier with resistor divider network and transistor linearizer network. The class-C amplifiers are working for high-voltage environments such that power resistors, electrolytic capacitors, and high-power choke inductors were used. Cooling fan system noises may affect the performance of the portable ultrasound instruments and ultrasound probes have limited structures and sizes to be contained with cooling fan systems. Additionally, class-C amplifiers have low heat [16]. Therefore, cooling fan systems might be used in addition to the 1-cm^2^ heat sinks attached to the top of the power MOSFETs for the experimental measurements.

**Figure 3:**
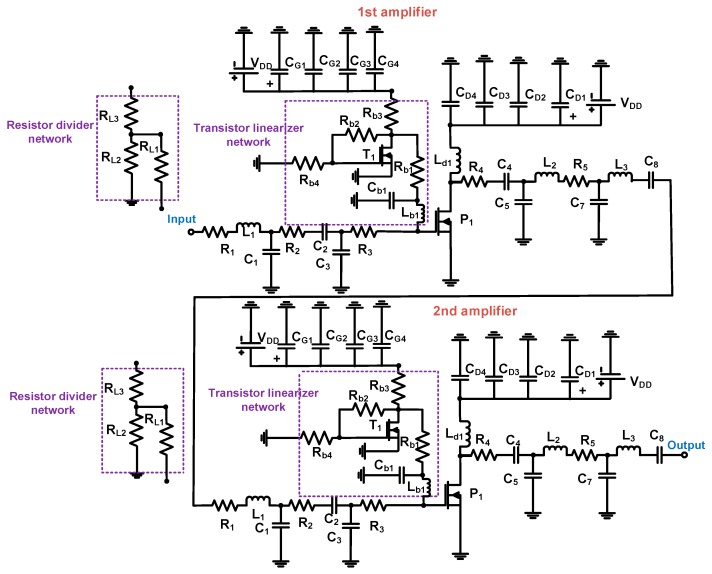
Schematic diagrams of class-C amplifiers with resistor divider network and transistor linearizer network.

Figure 3 shows the schematic diagrams of the class-C amplifier with resistor divider network and transistor linearizer network. The class-C amplifiers were composed of the two-stage amplifiers. In Figure 3, the typical resistor divider network was composed of resistors (R_L2_ and R_L3_). The resistor (R_L1_) was used for blocking the alternating current (AC) signals from the input port. The shunt choke inductor (L_d1_) was used to minimize the DC voltage drop, as the class-C amplifiers had low maximum output voltage swing. The electrolytic capacitors (C_G1_ = 10 μF and C_D1_ = 220 μF) with three additional capacitors (C_G2_ = C_D2_ = 0.1 μF, C_G3_ = C_D3_ = 1000 pF, and C_G4_ = C_D4_ = 47 pF) were used to reduce the noise signals from DC power supplies. Power MOSFETs (PD57018, STMicroelectronics, Geneva, Switzerland) were used because the operating frequency and drain–source voltage ranges of the power MOSFET are 1 GHz and 65 V, respectively. All electronic components were guaranteed to work under a high-voltage environment in the printed circuit board. 

In Figure 3, a transistor divider network, instead of a resistor divider network, was used to improve the bias voltage conditions for the class-C amplifier. The transistor linearizer network was designed to handle large voltage amplitudes from the input port of the amplifier, as the class-C amplifier has low DC operating point, resulting in reduced output voltage amplitudes with low input signal amplitudes. This phenomenon is undesirable for the piezoelectric transducers with low sensitivity in the portable ultrasound instruments [7]. Therefore, the large-signal pulsed-sinusoidal inputs are needed for class-C amplifier used such that it could affect the DC bias voltages because amplified large signals on the drain-source voltages of the power MOSFETs (P_1_ and P_2_) could reduce the maximum allowances of the gate–source voltages of the power MOSFETs [21]. Therefore, the linearizer circuit is needed to improve the linearity performance of the amplifiers.

**Figure 4:**
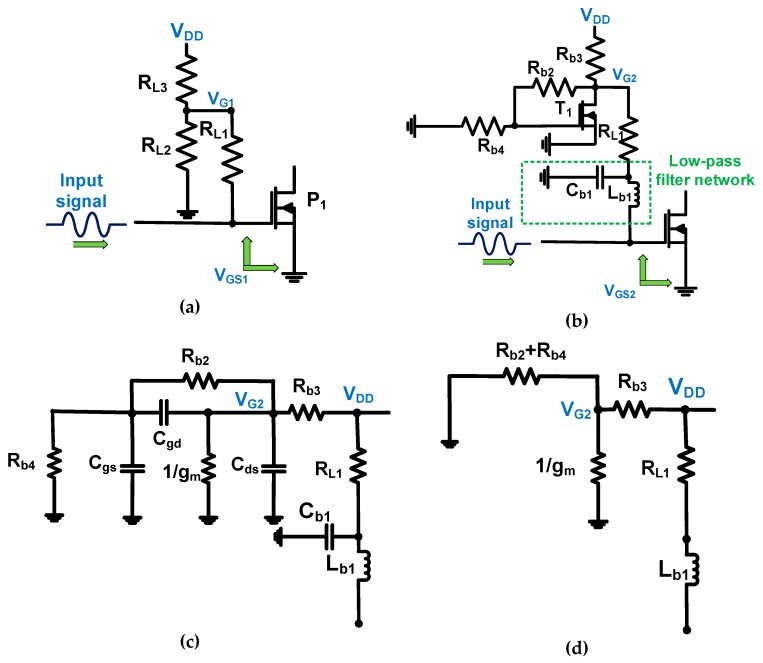
Schematic diagrams of (**a**) resistor divider network and (**b**) transistor linearizer network with main transistor (P_1_ and P_2_) of the class-C amplifiers. (**c**) Equivalent circuit model of the transistor linearizer network; (**d**) simplified equivalent circuit model of the transistor linearizer network without capacitors.

Figure 4 describes the operating mechanism of the resistor divider and transistor linearizer networks for class-C amplifiers. In the designed class-C amplifiers, the DC bias circuits require the amplifiers to be capable of sustaining an output voltage because the large-signal input voltage amplitudes up to 5 V_p-p_ is used as an input signal. As shown in Figure 4a, the DC bias voltage is defined as:

(1) $$V_{G1}=\frac{R_{L2}}{R_{L2}+R_{L3}}V_{\mathrm{DD}}$$

where V_DD_ is the supply voltage of the class-C amplifier and class-C linearized amplifier.

The large input signal can be passed through the blocking resistor (R_L1_) such that large-signal input signals around 5 V_p-p_ could affect the DC bias voltages of the class-C amplifier. Additionally, the resistance values (R_L2_ and R_L3_) in the resistor divider network are dependent on the temperature variance. Therefore, the resistor divider network might be undesirable when using large-signal input signals around 5 V_p-p_ for class-C amplifiers. This is because developed class-C amplifiers might have cooling fan systems for the portable ultrasound instruments. Additionally, the hot-carrier injection could make it difficult to maintain constant DC bias voltages for the amplifier because large input signals are sensitive to power MOSFET devices [22].

In Figure 4b, the high frequency and large input signal could be filtered out by the low-pass filter network, which is composed of the 120 pF capacitor (C_b1_) and 1 μH inductor (L_b1_). The cut-off frequency of this low-pass filter network is defined as:

(2) $$f_{c1}=\frac{1}{2\pi \sqrt{C_{b1}L_{b1}}}$$

The calculated and measured cut-off frequencies of the low-pass filter were 14.54 MHz and 14.15 MHz, respectively, and the input signals are 25 MHz, such that input signals above 15 MHz could be suppressed in the transistor linearizer network. The resistance values (R_b2_ and R_b4_) were selected to be higher than the parasitic impedances of the power MOSFET (T_1_, SQ2318AES-T1, Vishay Intertechnology, Malven, PA, USA) to reduce the undesirable parasitic effects and provide a DC bias voltage. Figure 4c shows the equivalent circuit model of the transistor linearizer network using the large signal power MOSFET library model [24]. The parasitic resistances and inductances of the power MOSFET could be removed because they were small values [24]. Figure 4d shows the simplified equivalent circuit model of the transistor linearizer network. Because the inverse value of the transconductance of the power MOSFET (1/g_mT1_) was smaller than the combined resistances (R_b2_ and R_b4_), the DC bias voltage for transistor linearizer network (V_G2_) is simplified as follows.

(3) $$V_{G2}=\frac{\left(\frac{1}{g_{mT1}}\right)//\left(R_{b2}+R_{b4}\right)}{\left(\frac{1}{g_{mT1}}\right)//\left(R_{b2}+R_{b4}\right)+R_{b3}}V_{DD}=\frac{1}{1+\frac{R_{b3}}{\left(\frac{1}{g_{mT1}}\right)//\left(R_{b2}+R_{b4}\right)}}V_{DD}\approx \frac{1}{1+g_{mT1}R_{b3}}V_{DD}$$

where g*_mT_*_1_ is the transconductance value of the power MOSFET (*T*_1_).

The DC bias voltages were dependent on the transconductance (g*_mT_*_1_) of the power MOSFET and resistance (R_b3_). Additionally, the power MOSFET transconductance was relatively less dependent on temperature variances and was constant with large signal inputs [25]. In the resistor divider network, two resistors (R_L2_ and R_L3_) could be sensitive to temperature variances because the temperature variances were dependent on the resistance values. In the transistor linearizer network, one resistor could be sensitive to temperature variances. Compared with the resistor divider network, the transistor linearizer network might be less susceptible to temperature variances. Therefore, the transistor divider network might be stable for temperature variances caused by large input signals. 

To reduce the temperature variances caused by the large input signals, a digitally programmed lookup table memory using analog to digital converter and analog to digital convert electronics is another solution [26,27]. However, these electronics are not desirable when using array transducers for portable ultrasound instruments as they are bulky and power consuming; instruments with limited size and architecture are preferred instead. For the amplifier measurement, the initial measured DC bias voltages for the resistor divider network and transistor linearizer network were initially both 2.70 V for the class-C amplifiers. As described in the introduction section, expected, and simulated data of the amplifier does not contain the accurate temperature parameters such that the measured bias voltages of the class-C amplifier and class-C linearized amplifiers for each hour were presented in Table 1.

These amplifiers were implemented to reduce the system sizes for portable ultrasound instruments. Therefore, the temperature dependences would affect the performances of the class-C amplifiers. The DC bias voltages of the class-C amplifier were reduced from 2.70 V and 2.41 V. The DC bias voltages of the class-C linearized amplifier were reduced from 2.70 V and 2.67 V. Therefore, the class-C linearized amplifier was less dependent with temperatures. In the result section, all the amplifier performances were measured just after 4 hr. Because of higher DC bias voltages the voltage gains and power consumptions could be expected to have higher values for class-C linearized amplifier.

Compared to the class A amplifiers, the class C amplifiers generate the output currents during on and off transition periods [16]. In addition, the output current and conduction angles have different values for class C amplifier and class C linearized amplifiers. Therefore, we needed to consider the different conduction angle and the output current values to calculate the voltage gain and DC power consumption for class C amplifier and class C linearized amplifier. Therefore, the output peak-to-peak voltage for the amplifiers can be represented as [11,16,23]:

(4) $$V_{\mathrm{out},\mathrm{pp}}=i_{\mathrm{out}}\cdot \frac{R_{\mathrm{load}}}{2\pi }\left(2\theta -\sin2\theta \right)$$

where i_out_ is the output current, R_load_ is the load resistance, and θ is the conduction angle of the class C amplifiers.

The voltage gain of the class C amplifiers (G) can be represented as the output peak-to-peak voltage divided by the input peak-to-peak voltage:

(5) $$G=20\log_{10}(i_{\mathrm{out}}\cdot \frac{R_{\mathrm{load}}}{2\pi \cdot V_{\mathrm{in},\mathrm{pp}}}\left(2\theta -\sin2\theta \right))$$

The DC power consumption (P_DC_) for the class C amplifier and class C linearized amplifier are represented as [16,18,22]:

(6) $$P_{\mathrm{DC}}=V_{\mathrm{DD}}\cdot \frac{i_{\mathrm{out}}}{\pi }\cdot \left(\sin\theta -\theta \cos\theta \right)$$

For the amplifiers, the values of the outputs (i_out_) and conduction angles (θ) in the DC power consumption are different. The power MOSFET transistor library provided from the manufacturer was inaccurate under high-voltage environments to generate the theoretical model parameter such that the measured performances were presented to characterize the class C amplifier and class C linearized amplifier. 

## 3. Results

The performance of the class-C amplifiers with resistor divider and transistor linearizer network need to be checked because small-sized piezoelectric transducers for portable ultrasound instruments have low sensitivity [28]. The class-C amplifier was designed for portable ultrasound instruments such that voltage gain and DC power consumption were evaluated. Class-C amplifiers are designed to reduce unwanted heat; therefore, the tests were carried out. Considering the temperature variance effects, all the performance of the class-C amplifiers were also measured.

**Figure 5:**
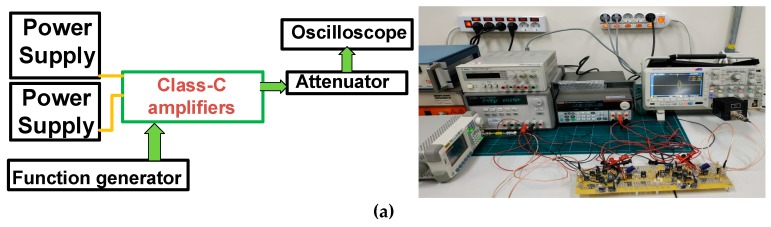

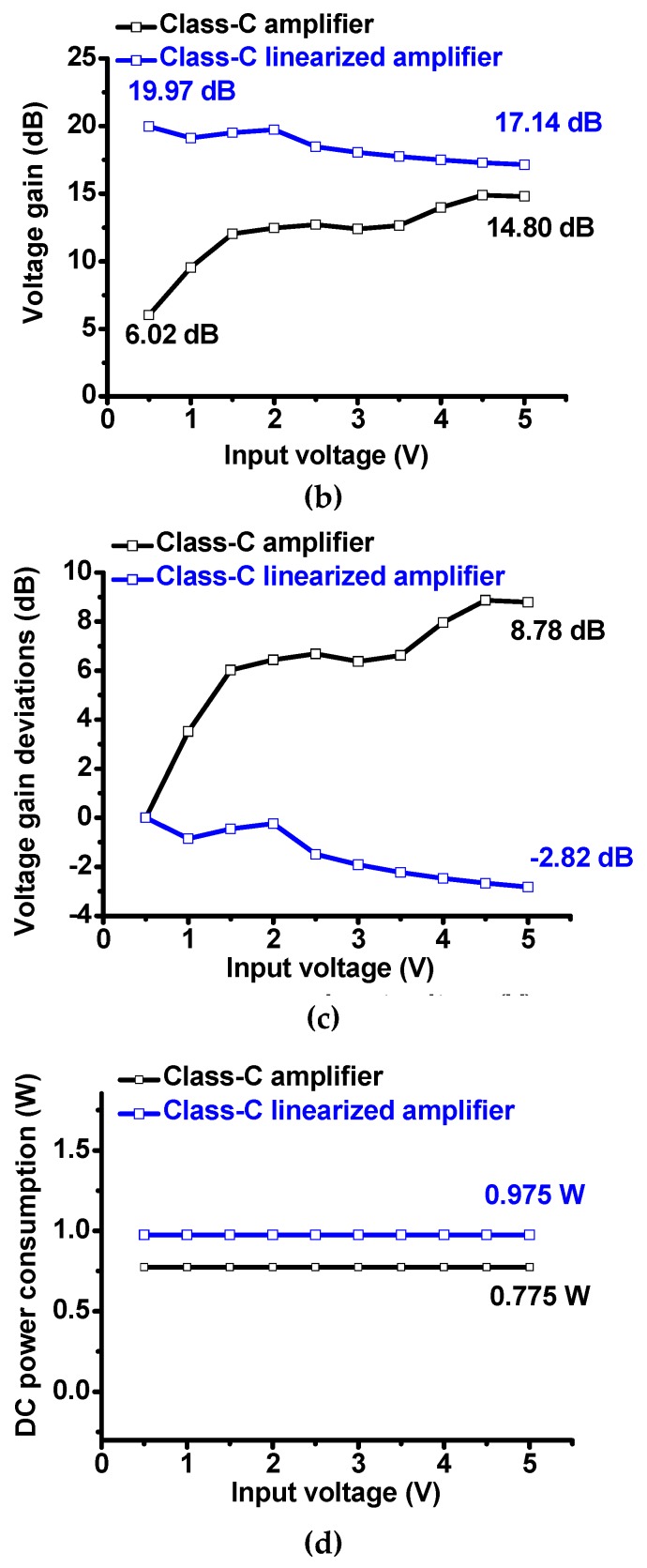
(**a**) Schematic diagram and photo of measurement setup; (**b**) voltage gain versus input peak-to-peak voltage amplitudes of class-C amplifier and class-C linearized amplifier; (**c**) voltage gain deviations vs. input peak-to-peak voltage amplitudes of class-C amplifier and class-C linearized amplifier; (**d**) DC power consumption versus input peak-to-peak voltage amplitudes of class-C amplifier and class-C linearized amplifier.

Figure 5a shows the measurement setup and its photo for the voltage gains, gain deviations, and DC power consumption versus input voltages. One power supply and another power supply provided the gate–source DC bias voltage of the class-C amplifiers. Function generator (DG5071, Rigol Technologies, Beijing, China) fed the pulsed sinusoidal waveforms up to 5 V_p-p_ into the designed class-C amplifiers. The amplified signals were reduced using attenuators and were recorded in the oscilloscope (MSO2024B, Tecktronics Inc., Beaverton, OR, USA). 

Figure 5b–d show the voltage gain, gain deviation, and DC power consumption versus input voltage of the class-C amplifier and class-C linearized amplifier, respectively. As shown in Figure 5b, the lowest voltage gain of the class-C linearized amplifier (17.14 dB) was still higher than the maximum voltage gain of the class-C amplifier (14.80 dB). Therefore, the measurement data could confirm that the transistor linearizer network was less dependent on temperature variances such that the linearizer can increase voltage gain for same input voltages. In Figure 5c, the absolute value of the voltage gain deviation of the class-C linearized amplifier (−2.82 dB) is still lower than that of the class-C amplifier (8.78 dB) at 5 V_p-p_ input voltage. Therefore, the linearity of the class-C amplifier is improved by the linearizer. In Figure 5d, the DC power consumption of the class-C linearized amplifier (0.975 W) is slightly higher than that of the class-C amplifier (0.775 W). However, the DC power consumption of each amplifier is lower than 1 W; thus, both could be useful for portable ultrasound instruments.

**Figure 6:**
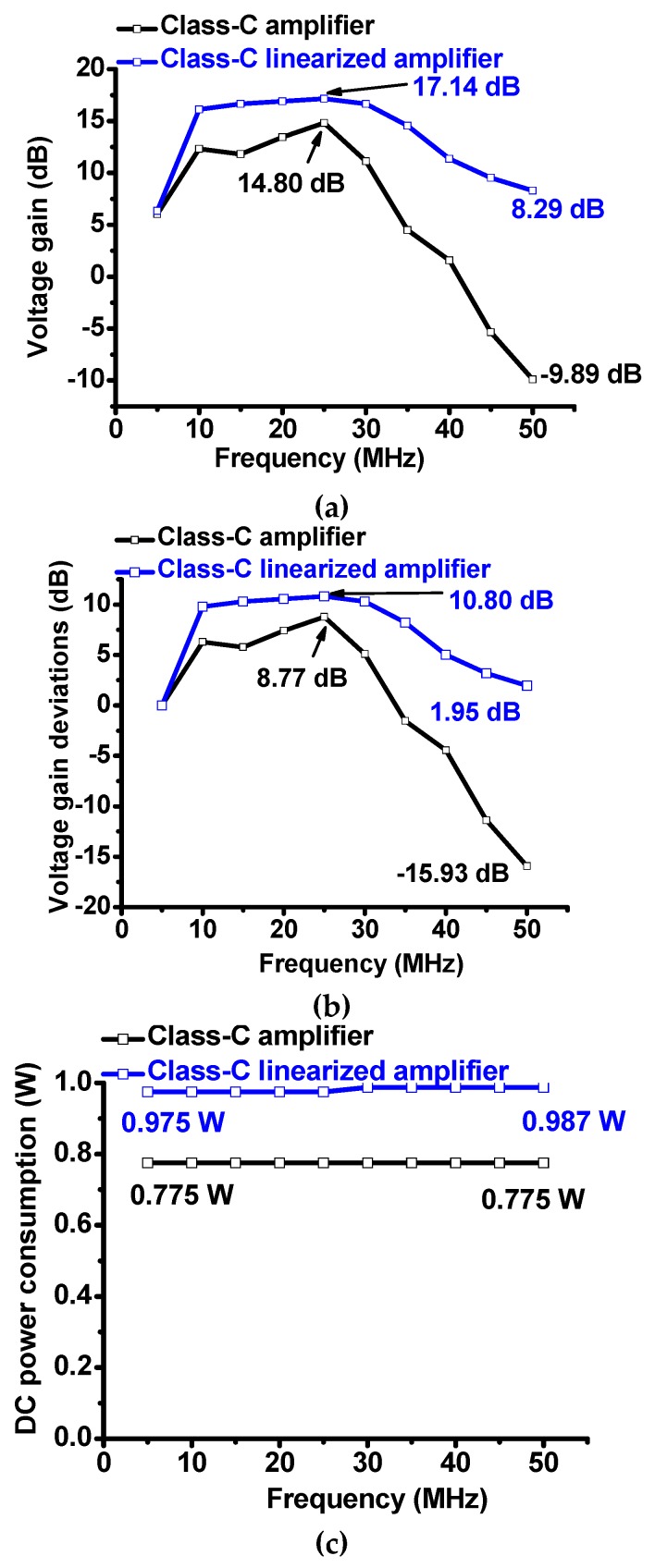
(**a**) Voltage gains versus frequencies of class-C amplifier and class-C linearized amplifier; (**b**) voltage gain deviations versus frequencies of class-C amplifier and class-C linearized amplifier; (**c**) DC power consumption versus frequencies of class-C amplifier and class-C linearized amplifier.

Figure 6 shows the measurement results of the voltage gain, gain deviations, and DC power consumption versus frequencies of the class-C amplifier and class-C linearized amplifier, respectively, because some piezoelectric transducers have relatively wide bandwidths. As shown in Figure 6a, the maximum voltage gains of the class-C amplifier and class-C linearized amplifier were 14.80 dB and 17.14 dB, respectively. As shown in Figure 6b, the maximum voltage gain deviation of the class-C linearized amplifier (1.95 dB) is lower than that of the class-C amplifier (−15.93 dB) at 50 MHz. Therefore, the transistor linearizer can also improve the linearity performance of the class-C amplifier with less temperature dependence for wide frequency ranges. 

As shown in Figure 6c, the DC power consumption of the class-C amplifier is constant between 10 MHz and 50 MHz (0.775 W). The DC power consumption of the class-C linearized amplifier at 50 MHz (0.987 W) is a little bit higher than that of the class-C amplifier at 50 MHz (0.975 W). However, the DC power consumption between 10 MHz and 50 MHz is still lower than 1 W, such that the class-C amplifiers are still useful to reduce the DC power consumption for the portable ultrasound instruments. However, higher frequency and higher input voltages, resulting in increasing the temperatures were used for designed class C amplifiers because of lower voltage gains. These configuration increases the temperatures of the power MOSFETs and power resistors such that they can reduce the performances of the class C amplifiers as frequency increases as shown in Figure 6.

The theoretical equations of the class C amplifiers were presented. However, the theoretical and modeled data of the amplifiers are unpredictable when using the electronic amplifier devices for high-voltage environments, because the power MOSFETS do not have signal-distortion accuracy even for the sub-decibel levels [21]. Additionally, the temperature model parameters for power MOSFFETs in the simulation model are inaccurate under high-voltage environments [22]. Therefore, pulse-echo measurements were performed to evaluate the characteristics of the amplifier. 

**Figure 7:**
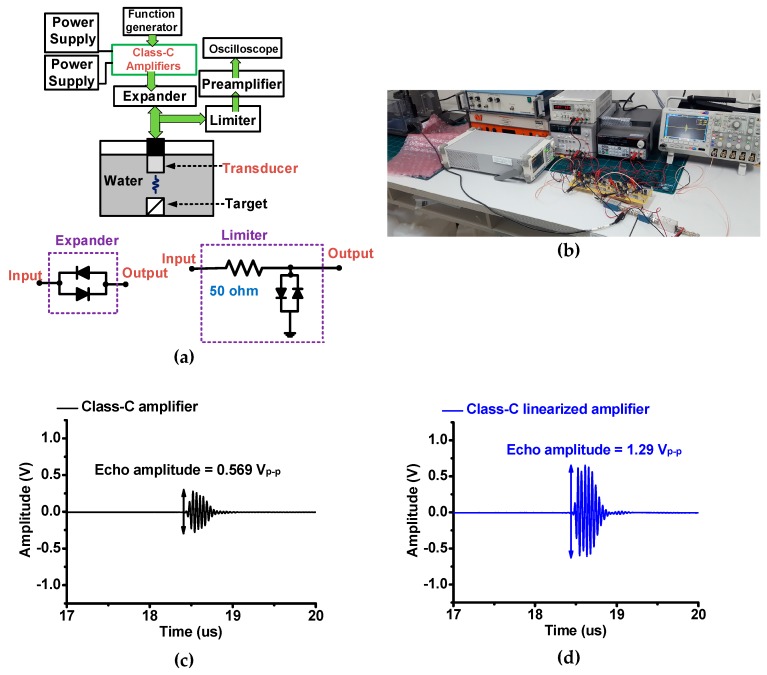

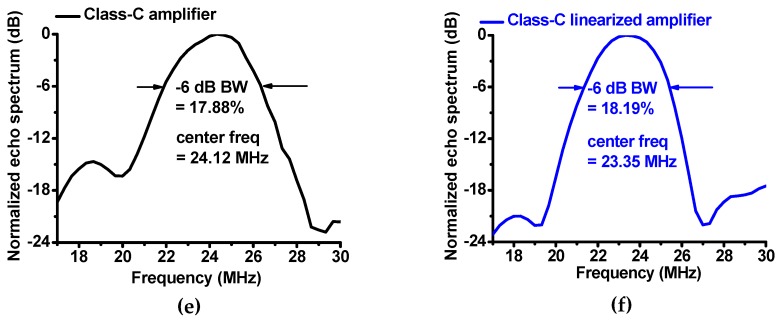
(**a**) Schematic diagram and (**b**) photo of the pulse-echo measurement; echo signal amplitudes generated by piezoelectric transducer when using (**c**) class-C amplifier and (**d**) class-B linearized amplifier. Normalized spectrum of the echo signal amplitudes generated by the piezoelectric transducer when using (**e**) class-C amplifier and (**f**) class-C linearized amplifier. BW and center freq represent the –6 dB bandwidth and center frequency of the echo signals, respectively.

Figure 7 shows typical pulse-echo measurement setup to evaluate class-C amplifier performance with a piezoelectric transducer [29]. The five-cycle sinusoidal waveforms generated from the function generator (DG5071) were fed into the class-C amplifiers with DC bias voltages provided from DC power supplies. The expander circuit was constructed to reduce the fluctuation of the amplified signals generated from the amplifiers. The limiter circuit was used to protect the preamplifier (AU-1525, L3 NARDA-MITEQ Inc., Hauppauge, NJ, USA) and oscilloscope (MSO2024B). The amplified sinusoidal waveforms trigger the focused transducer (Olympus NDT, Waltham, MA, USA) to generate the acoustic waves. The transmitted acoustic waveforms were reflected from the target. The reflected acoustic waveforms were converted by the piezoelectric transducer into electrical waveforms while the discharged electrical sinusoidal waveforms were suppressed by the limiter circuit. The received electrical waveforms by the piezoelectric transducer were amplified by a preamplifier and recorded in the oscilloscope.

The echo amplitude when using the class-C linearized amplifier (1.29 V_p-p_) was higher than that when using the class-C amplifier (0.569 V_p-p_). The −6 dB bandwidth of the normalized echo spectrum when using class-C linearized amplifier is similar (18.19%) to that when using the class-C amplifier (17.88%). The center frequency of the normalized spectrum when using the class-C linearized amplifier is still similar (23.35 MHz) to that when using class-C amplifier (24.12 MHz). Therefore, the linearizer circuit can increase the sensitivity of the piezoelectric transducer, which is useful for portable ultrasound instruments because the miniaturized array-type transducer typically generates low sensitivity.

## 4. Conclusions

Class-C amplifier is one of the most efficient amplifiers, thus, it is able to enhance the battery life of portable ultrasound instruments. However, reduced linearity of the class-C amplifier could affect the sensitivity of the piezoelectric transducer, which is main component of the instrument; this could be a critical issue because the echo signals generated by the piezoelectric transducers are relatively low. Therefore, a class-C amplifier transistor linearizer scheme was proposed for the instruments to increase sensitivity while maintaining low DC power consumption and reducing unwanted heat.

Compared with the resistor divider network, the transistor linearizer network for class-C amplifiers could effectively reduce the large pulsed sinusoidal signals using a low-pass filter network and provide a stable DC bias voltage with respect to the temperature variances. To reduce the temperature variances caused by large input signals, the DC bias adjustment using a programmed lookup table memory, analog-to-digital converter, and digital-to-analog converter could be another solution. However, it is not suitable for piezoelectric transducers in portable ultrasound instruments because these electronics are bulky and power-consuming devices. 

To verify the capability of the transistor linearizer network for the class-C amplifiers, the voltage gain and DC power consumption versus input voltage amplitudes were measured. The measured voltage gain of the class-C amplifier and class-C linearized amplifier were 14.80 dB and 17.14 dB, respectively. Thus, compared with class-C amplifier, class-C linearized amplifier could obtain higher voltage gain for wide input voltage ranges, thus, improving sensitivity performance of the piezoelectric transducers. The DC power consumption of the class-C amplifier and class-C linearized amplifiers were 0.775 W and 0.975 W, respectively. However, the DC power consumption of both amplifiers is still less than 1 W. 

To be capable of the suitability for the portable ultrasound instruments, the performance of the class-C amplifiers with a resistor divider and transistor linearizer networks were tested. The echo amplitude when using the class-C linearized amplifier (1.29 V_p-p_) was improved compared with that when using the class-C amplifier (0.569 V_p-p_). However, the −6 dB bandwidth of the normalized echo spectrum when using class-C amplifier (17.88%) is similar compared to that when using class-C linearized amplifier (18.19%). Additionally, the center frequency of the normalized spectrum when using class-C amplifier is still similar (24.12 MHz) compared to that when using class-C linearized amplifier (23.35 MHz). Therefore, improved sensitivity could be beneficial for portable ultrasound instruments, owing to the higher voltage gains of the class-C linearized amplifier. 

## Figures and Tables

**Table 1:** The DC bias voltages of the class-C amplifier and class-C linearized amplifier.

Duration	Class-C Amplifier		Class-C Linearized Amplifier	
	Calculated	Measured	Calculated	Measured
8 min	2.90 V	2.70 V	2.90 V	2.70 V
2 h	2.90 V	2.60 V	2.90 V	2.69 V
3 h	2.90 V	2.52 V	2.90 V	2.68 V
4 h	2.90 V	2.41 V	2.90 V	2.67 V
